# Development of ceiling board using breadfruit seed coat and recycled low density polyethylene

**DOI:** 10.1016/j.heliyon.2019.e02712

**Published:** 2019-11-14

**Authors:** O.N. Ezenwa, E.N. Obika, C. Umembamalu, F.C. Nwoye

**Affiliations:** a Department of Mechanical Engineering, Nnamdi Azikiwe University, Awka, Anambra State, Nigeria; b Department of Chemical Engineering, Nnamdi Azikiwe University, Awka, Anambra State, Nigeria; c Department of Mechanical Engineering, Akanubian Federal Polythechnic, Uwana, Ebonyi State, Nigeria

**Keywords:** Agricultural science, Chemistry, Environmental science, Materials science, Bread fruit seed, Ceiling board, Polyethylene, Agro waste, Filler material

## Abstract

Conversion of agro wastes into potential raw materials for production has gained a lot of attention in research. This has led to the development of ceiling board using waste breadfruit seed coat and Recycled Low Density Polyethylene, as filler and binder respectively. The filler material (Breadfruit seed coat), was treated with 1 mol/dm^3^ of NaOH to eliminate the pigment and neutralized using 0.5 mol/dm^3^ Acetic Acid. After drying, the filler material was ground to a particle size of 600μm. Using Central Composite Design (CCD) tool of the Design Expert software, the experimental design was set up. From the design, thirty experimental samples were developed with the production parameters; press time, press pressure, press temperature and filler/rLDPE ratio as the independent variables. The produced samples were tested for Thermal Conductivity, Water Absorption, Thickness Swell and Density tests, which formed the responses for the Central Composite Design. The results of the experiment were analysed using the Response Surface Methodology while the validation was done using the Analysis of Variance (ANOVA). The optimal values obtained for the production parameters are 19.722% filer/rLDPE, 10minutes press time, 197.31 °C press temperature and 9.042MPa press pressure, which gave a 775.661 g/cm^3^ density, 0.308% Water Absorption, 0.962% Thickness Swell and 0.367W/M.K Thermal Conductivity. This result shows that agro waste breadfruit seed coat is a good filler material for the production of ceiling board.

## 1 Introduction

The dominance of peasant farming in most developing countries, explains the abundance of agricultural and natural fibres in these countries since they produce a vast range of agricultural products like palm trees, rice, sugarcane and the rest of other crops (Akinyemi et al., 2016). The agricultural wastes generated in the production of these crops are mostly used as fuel or burnt off on disposal sites thereby constituting environmental hazards such as emission of CO_2_, one of the gases responsible for global warming and degradation of agricultural soil. Research has shown that these natural fibres have very good physical and mechanical properties hence, making them potential raw materials for various building applications (Suleiman et al., 2013).

Organic fibres play a vital role in solving the problems associated with construction materials. They are readily available in most parts of the world, and can also reduce the consumption energy. The use of these natural organic fibres help to conserve the limited available materials as well as environmental protection hence, they have an important part to play in ecological cycle. Wastes such as of vegetables, food products, cotton stalk, sugarcane bagasse, paddy and wheat straw and husk, jute fiber, groundnut shell, wooden mill waste, coconut husk, etc, constitute a greater portion of the wastes generated from agricultural sources (Akinyemi et al., 2016). The utilization of locally obtainable agro waste materials have been considered and reviewed depending on the required end product construction material (viz. particle boards, thermal insulators, masonry composites/bricks, cementations/binder, aggregates, etc.) (Amit and Ipshita, 2015). Works on ceiling board using agro-wastes include the use of rice husk (Oladele et al., 2009; Suleiman et al., 2013; Madu et al., 2018), banana fibres (Stephen et al., 2014), jatropha curcas seedcake material (Olorunmaiye and Ohijeagbon, 2015), water melon peels (Idris et al., 2011), bamboo (Chibudike et al., 2011), corn cobs and cassava stalks (Amenaghawon et al., 2016) as well as other synthetic wastes like sawdust (Idehai, 2012; Akinyemi et al., 2016; Atuanya and Obele, 2016; Isheni et al., 2017; Olufemi et al., 2012) and waste paper (Ekpunobi et al., 2015). Despite the fact that a lot of agro-wastes have been utilized in the production of ceiling board, the utilization of bread fruit seed coat for ceiling board production is not available in literature, hence it forms the knowledge gap this research aims to fill. The optimisation tool of the Design expert software was used to optimize both the production processes and the percentage composition of the filler and binder. This gave an interesting optimal values of 19.722% filer/rLDPE, 10minutes press time, 197.31 °C press temperature and 9.042MPa press pressure. This will result to 775.661 g/cm^3^ density, 0.308% Water Absorption, 0.962% Thickness Swell and 0.367W/M.K Thermal Conductivity, that competes favorably with existing ceiling boards.

## 2 Materials and methods

The materials used for this work were; Recycled low density polyethylene, bread fruit coat, water, sodium hydroxide (NaOH), acetic acid (Achebe et al., 2019). The materials as well as their sources are shown in Table 1.

**Table 1:** Materials and their sources.

S/N	Materials	Source
1	Recycled Low Density Polyethylene (rLDPE)	Obtained from a vendor in Onitsha, Anambra State, Nigeria.
2	Bread fruit seed	Obtained from a vendor in Amansea, Anambra State, Nigeria.
3	NaOH	Pure Chemicals Co., Anna Nagar, Chennai, India
4	Acetic Acid	Pure Chemicals Co., Anna Nagar, Chennai, India

### 2.1 Preparation of bread fruit coat

Breadfruit coats were collected from a breadfruit seed dealer in Amansea, Anambra State. The coats were suspended in a 1 mol/dm^3^ solution of NaOH for one hour to remove the pigment. After washing out the NaOH, the coats were neutralized using 0.5 mol/dm^3^ of acetic acid. The coats were then washed with water to remove the acetic acid and sun dried. The dried coats was ground into powder and sieved to 600μm particle size.

### 2.2 Preparation of low density polyethylene

The Low density polyethylene was obtained from a recycling company in Onitsha. They were recycled and processed into pellet forms. On purchase, the recycled low density polyethylene was ground into powdery form and sieved to 600μm particle size.

### 2.3 Development of samples

**Fig. 1:**
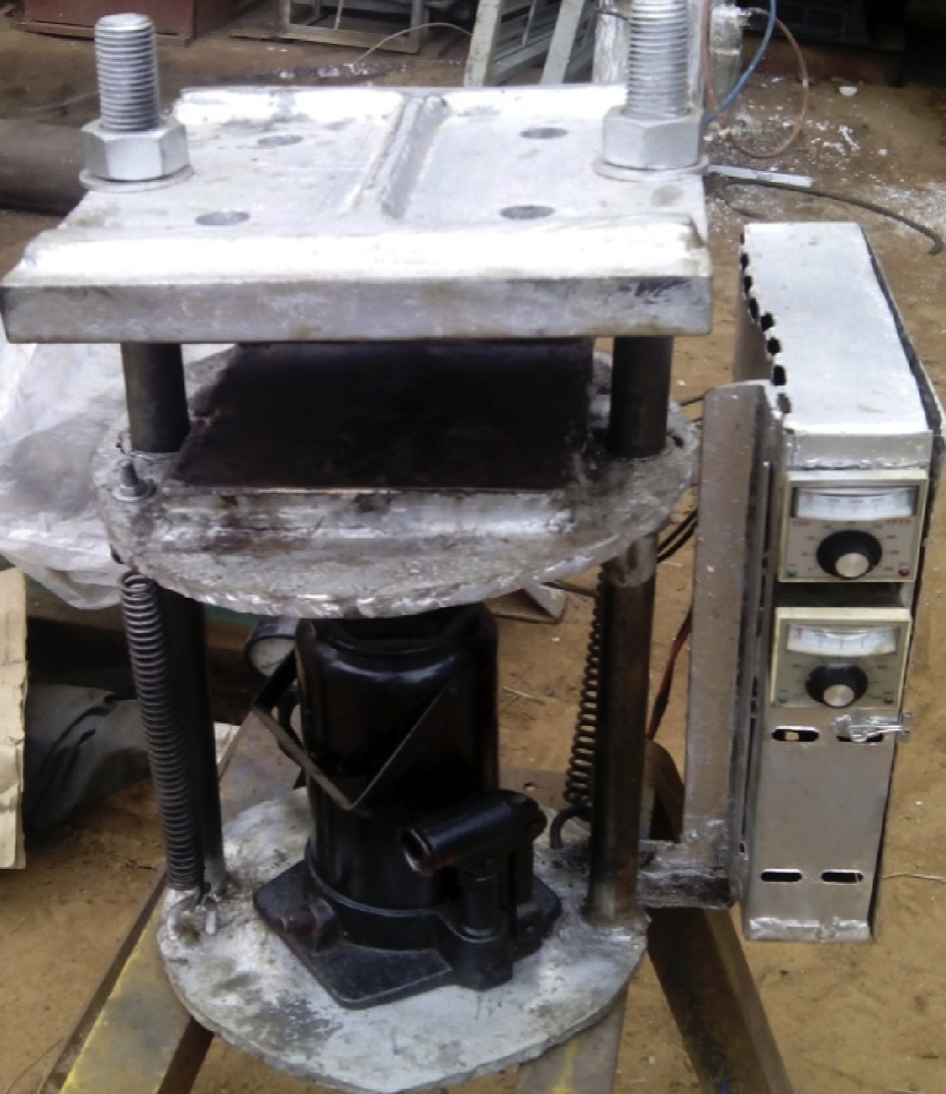
Constant temperature hydraulic press.

The weighed contents samples of the filler material (Bread fruit seed coat) and binder (recycled low density polyethylene), as shown in Table 2, were put in a bowl and manually dry-mixed by the use of stirring rod until a homogeneous mixture was obtained. The mixtures of the filler and binder for each sample were separately transferred into the rectangular mold. The mold containing the mixed materials was pressed at various press time and pressure using the constant temperature hydraulic press as stipulated in the sample design(see Fig. 1).

**Table 2:** Experimental Setup for 5level-Four factorial response surface design for Ceiling board.

Std	Filler/rLDPE wt %	B:Press time (min)	C:Press Temp. (°C)	D:Press pressure (MPa)	Density Kg/cm^3^	Water absorption %	Thickness Swell %	Thermal Conductivity W/M.K
1	10	5	180	7	742	1.74	9.34	0.98
2	20	5	180	7	612	22.9	6.19	0.95
3	10	10	180	7	633	7.88	10.1	0.83
4	20	10	180	7	700	10.9	0.91	0.65
5	10	5	200	7	656	1.43	16.1	0.38
6	20	5	200	7	598	8.75	16.0	0.43
7	10	10	200	7	766	7.95	12.9	0.49
8	20	10	200	7	851	0.41	10.3	0.36
9	10	5	180	11	772	1.93	10.2	0.58
10	20	5	180	11	706	24.8	14.7	0.57
11	10	10	180	11	591	7.52	17.1	0.42
12	20	10	180	11	643	12.4	8.44	0.49
13	10	5	200	11	722	0.36	8.97	0.36
14	20	5	200	11	660	8.47	9.65	0.42
15	10	10	200	11	733	0.96	11.1	0.45
16	20	10	200	11	761	0.31	2.87	0.52
17	5	7.5	190	9	625	3.06	1.65	0.59
18	25	7.5	190	9	592	19.8	1.97	0.49
19	15	2.5	190	9	608	8.18	1.41	0.66
20	15	12.5	190	9	656	2.26	2.09	0.50
21	15	7.5	170	9	599	17.5	1.98	0.84
22	15	7.5	210	9	719	0.53	7.37	0.39
23	15	7.5	190	5	893	4.98	26.6	0.64
24	15	7.5	190	13	897	3.70	29.8	0.39
25	15	7.5	190	9	742	3.03	3.27	0.38
26	15	7.5	190	9	772	2.95	7.23	0.29
27	15	7.5	190	9	786	3.25	7.23	0.26
28	15	7.5	190	9	798	2.85	7.01	0.31
29	15	7.5	190	9	745	4.75	8.03	0.37
30	15	7.5	190	9	755	2.45	7.46	0.19

### 2.4 Optimization of the production parameters

The production process was optimized using Response surface methodology, with the independent variables being press time, press temperature, press pressure and fiber to binder (filler/rLDPE) ratio, as shown in Table 3. This means that both the production process and material mixture ratio will be optimized.

**Table 3:** Factor Levels of independent variables.

Factor	Name	Units	Minimum	Maximum	Low	High	Mean
A	Filler/rLDPE	wt %	5.00	25.0	10.0	20.0	15.0
B	Press time	(min)	2.50	12.5	5.00	10.0	7.50
C	Press Temp.	(°C)	170	210	180	200	190
D	Press pressure	(MPa)	5.00	13.0	7.00	11.0	9.00

#### 2.4.1 Central Composite Design (CCD)

The production process was optimized using the Central Composite Design (CCD). The factors or independent variables considered were; press time, press temperature, press pressure and fiber to binder ratio, while the dependable variables or responses are, tensile strength, density, thickness swell, water absorption rate and thermal conductivity.

With the CCD, the variables were varied at five different levels (-α, -1, 0, 1, +α) giving a total of thirty (30) different experimental samples. Among these experimental samples, there were fourteen (14) core points, ten (10) star points and six (6) centre points. This gave two replicates for both the factorial points and axial (star) points each to increase the accuracy of the experiment. With this the experimental design was obtained as shown in Table 3.

### 2.5 Responses

They are also called the independent variable. They are the various properties (thermal resistance, density, water absorption and thickness swell) that are needed to define the quality of a ceiling board. These properties were obtained using the procedures described in below.

#### 2.5.1 Density

To determine the density of each sample the mass was first measured and recorded. Since the samples are rectangular in shape, the volume becomes the product of the length, width and thickness. Hence, the density of each sample is calculated by dividing its mass by the volume.

#### 2.5.2 Thickness swell

This response determines the degree of deformation (increase in thickness) that will occur when the material absorbs moisture. To perform this test, the inititial thickness (T_o_) is measured using the micro-meter screw gauge and recorded. The sample is then partially submerged into water up to a height of 20cm and allowed in that position for 24hours. After that, the thickness is measured and recorded as T_1_. The thickness swell is calculated as thus;

(1) $$Ts=\frac{T_{1}-T_{o}}{T_{o}}$$

#### 2.5.3 Water absorption

This is similar to that of thickness swell but in this case, it is the weight increase that is determined. This response will help determine the percentage increase in weight that will occur in a case of moisture absorption. Here, the initial and final weight of the material before and after submerging partially into water is measured and recorded as W_0_ and W_1_ respectively. The waters absorption rate by weight is given as;

(2) $$WA=\frac{W_{1}-W_{o}}{W_{o}}$$

#### 2.5.4 Thermal conductivity

Ceiling boards with lower thermal conductivity are chosen over those with relatively higher thermal conductivity, because heat resistivity is the property of a ceiling board that helps maintain the temperature of a room. The samples were placed between the cold and hot surfaces of the thermal conductivity testing machine. With T_o_ and T_1_ as the temperatures of the hot and cold surfaces at steady state, thermal flux of the heater ‘⍴’, surface area of sample ‘A’, thickness of sample ‘t’ the thermal conductivity K is calculated as thus;

(3) $$k=\frac{\rho t}{A\left(T_{o}-T_{1}\right)}$$

## 3 Results

### 3.1 Effect of factors on density

#### 3.1.1 ANOVA for density

The analysis of variance for quadratic model of density is given in Table 4 (see Tables 5, 6, 7, 8 and 9)

**Table 4:** Analysis of variance for Quadratic model of Density.

Source	Sum of Squares	df	Mean Square	F-value	p-value	
**Model**	2.144E+05	14	15315.09	42.82	<0.0001	significant
A-Filler/rLDPE	927.45	1	927.45	2.59	0.1282	
B-Press time	3888.41	1	3888.41	10.87	0.0049	
C-Press Temp.	14442.23	1	14442.23	40.37	<0.0001	
D-Press pressure	62.83	1	62.83	0.1756	0.6811	
AB	18682.03	1	18682.03	52.23	<0.0001	
AC	297.74	1	297.74	0.8324	0.3760	
AD	6.29	1	6.29	0.0176	0.8963	
BC	34188.31	1	34188.31	95.58	<0.0001	
BD	13930.21	1	13930.21	38.94	<0.0001	
CD	26.17	1	26.17	0.0731	0.7905	
A^2^	43262.05	1	43262.05	120.94	<0.0001	
B^2^	31388.57	1	31388.57	87.75	<0.0001	
C^2^	20270.16	1	20270.16	56.67	<0.0001	
D^2^	27780.90	1	27780.90	77.66	<0.0001	
**Residual**	5365.55	15	357.70			
Lack of Fit	2766.20	10	276.62	0.5321	0.8149	not significant
Pure Error	2599.35	5	519.87			
**Cor Total**	2.198E+05	29

**Table 5:** ANOVA for Quadratic model of Water Absorption.

Source	Sum of Squares	df	Mean Square	F-value	p-value	
**Model**	1305.67	14	93.26	62.64	<0.0001	significant
A-Filler/rLDPE	357.71	1	357.71	240.27	<0.0001	
B-Press time	47.80	1	47.80	32.10	<0.0001	
C-Press Temp.	378.19	1	378.19	254.02	<0.0001	
D-Press pressure	2.53	1	2.53	1.70	0.2124	
AB	223.01	1	223.01	149.79	<0.0001	
AC	124.45	1	124.45	83.59	<0.0001	
AD	7.88	1	7.88	5.29	0.0362	
BC	0.6741	1	0.6741	0.4528	0.5112	
BD	2.86	1	2.86	1.92	0.1863	
CD	8.48	1	8.48	5.70	0.0306	
A^2^	114.60	1	114.60	76.97	<0.0001	
B^2^	6.49	1	6.49	4.36	0.0543	
C^2^	56.45	1	56.45	37.92	<0.0001	
D^2^	1.96	1	1.96	1.32	0.2692	
**Residual**	22.33	15	1.49			
Lack of Fit	19.15	10	1.91	3.01	0.1179	not significant
Pure Error	3.18	5	0.6365			
**Cor Total**	1328.01	29

**Table 6:** ANOVA for Quadratic model Thickness Swell.

Source	Sum of Squares	df	Mean Square	F-value	p-value	
**Model**	1407.93	14	100.57	27.05	<0.0001	significant
A-Filler/rLDPE	12.45	1	12.45	3.35	0.0872	
B-Press time	2.02	1	2.02	0.5434	0.4724	
C-Press Temp.	39.51	1	39.51	10.63	0.0053	
D-Press pressure	11.54	1	11.54	3.10	0.0985	
AB	98.97	1	98.97	26.62	0.0001	
AC	0.4934	1	0.4934	0.1327	0.7207	
AD	2.09	1	2.09	0.5631	0.4646	
BC	21.83	1	21.83	5.87	0.0285	
BD	0.0205	1	0.0205	0.0055	0.9418	
CD	193.57	1	193.57	52.06	<0.0001	
A^2^	34.30	1	34.30	9.22	0.0083	
B^2^	35.19	1	35.19	9.47	0.0077	
C^2^	4.42	1	4.42	1.19	0.2928	
D^2^	827.14	1	827.14	222.46	<0.0001	
**Residual**	55.77	15	3.72			
Lack of Fit	41.00	10	4.10	1.39	0.3770	not significant
Pure Error	14.77	5	2.95			
**Cor Total**	1463.70	29

**Table 7:** ANOVA for Quadratic model Thermal Conductivity.

Source	Sum of Squares	df	Mean Square	F-value	p-value	
**Model**	1.06	14	0.0759	26.07	<0.0001	significant
A-Filler/rLDPE	0.0035	1	0.0035	1.20	0.2902	
B-Press time	0.0253	1	0.0253	8.68	0.0100	
C-Press Temp.	0.3611	1	0.3611	123.93	<0.0001	
D-Press pressure	0.1301	1	0.1301	44.64	<0.0001	
AB	0.0036	1	0.0036	1.23	0.2856	
AC	0.0026	1	0.0026	0.8967	0.3587	
AD	0.0151	1	0.0151	5.18	0.0379	
BC	0.0535	1	0.0535	18.35	0.0007	
BD	0.0089	1	0.0089	3.04	0.1017	
CD	0.1308	1	0.1308	44.88	<0.0001	
A^2^	0.0960	1	0.0960	32.95	<0.0001	
B^2^	0.1274	1	0.1274	43.74	<0.0001	
C^2^	0.1647	1	0.1647	56.53	<0.0001	
D^2^	0.0754	1	0.0754	25.89	0.0001	
**Residual**	0.0437	15	0.0029			
Lack of Fit	0.0185	10	0.0019	0.3686	0.9159	not significant
Pure Error	0.0252	5	0.0050			
**Cor Total**	1.11	29

**Table 8:** Constraints for optimization.

Name	Goal	Lower Limit	Upper Limit	Lower Weight	Upper Weight	Importance
A:Filler/rLDPE	is in range	10	20	1	1	3
B:Press time	is in range	5	10	1	1	3
C:Press Temp.	is in range	180	200	1	1	3
D:Press pressure	is in range	7	11	1	1	3
Density	is in range	591	897.01	1	1	5
WA	minimize	0.307692	24.79	1	1	5
TS	minimize	0.34	29.84	1	1	5
Thermal Conductivity	minimize	0.19	0.98	1	1	5

**Table 9:** Solutions of optimization.

Number	Filler/rLDPE	Press time	Press Temp.	Press pressure	Density	WA	TS	Thermal Conductivity	Desirability	
1	19.7	10.0	197	9.04	776	0.308	0.962	0.367	0.913	Selected
2	19.7	10.0	197	9.05	776	0.308	0.995	0.366	0.913	
3	19.7	10.0	197	9.05	776	0.308	0.938	0.367	0.913	
4	19.7	10.0	197	9.02	776	0.308	1.01	0.366	0.913	
5	19.7	10.0	197	9.07	775	0.308	0.923	0.368	0.913	
6	19.8	10.0	197	9.06	775	0.308	0.884	0.368	0.913	
7	19.6	10.0	197	9.05	776	0.308	1.07	0.364	0.913	
8	19.8	10.0	197	9.04	775	0.307	0.854	0.369	0.913	
9	19.7	10.0	197	9.01	777	0.308	1.07	0.364	0.913	
10	19.6	10.0	197	9.07	776	0.308	1.08	0.364	0.913	

The Model F-value of 42.82 implies the model is significant. There is only a 0.01% chance that an F-value this large could occur due to noise. P-values less than 0.0500 indicate model terms are significant. The Lack of Fit F-value of 0.53 implies the Lack of Fit is not significant relative to the pure error. There is an 81.49% chance that a Lack of Fit F-value this large could occur due to noise.

Considering the fact that the terms with P-Values less than 0.05 are the significant terms according to the Analysis of Variance (ANOVA), the final equation reduces to;

(4) Density = −6885.73902 − 252.92428X_2_ + 91.16929X_3_ + 2.73364X_1_X_2_ + 1.84901X_2_X_3_ - 5.90132X_2_X_4_ − 1.58859X_1_^2^ − 5.41258X_2_^2^ − 0.271849X_3_^2^ + 7.95631X_4_^2^

Where X_1_ = filler/RLDPE, X_2_ = Press time, X_3_ = Press temperature and X_4_ = Press pressure.

#### 3.1.2 3D surface plots for density

**Fig. 2:**
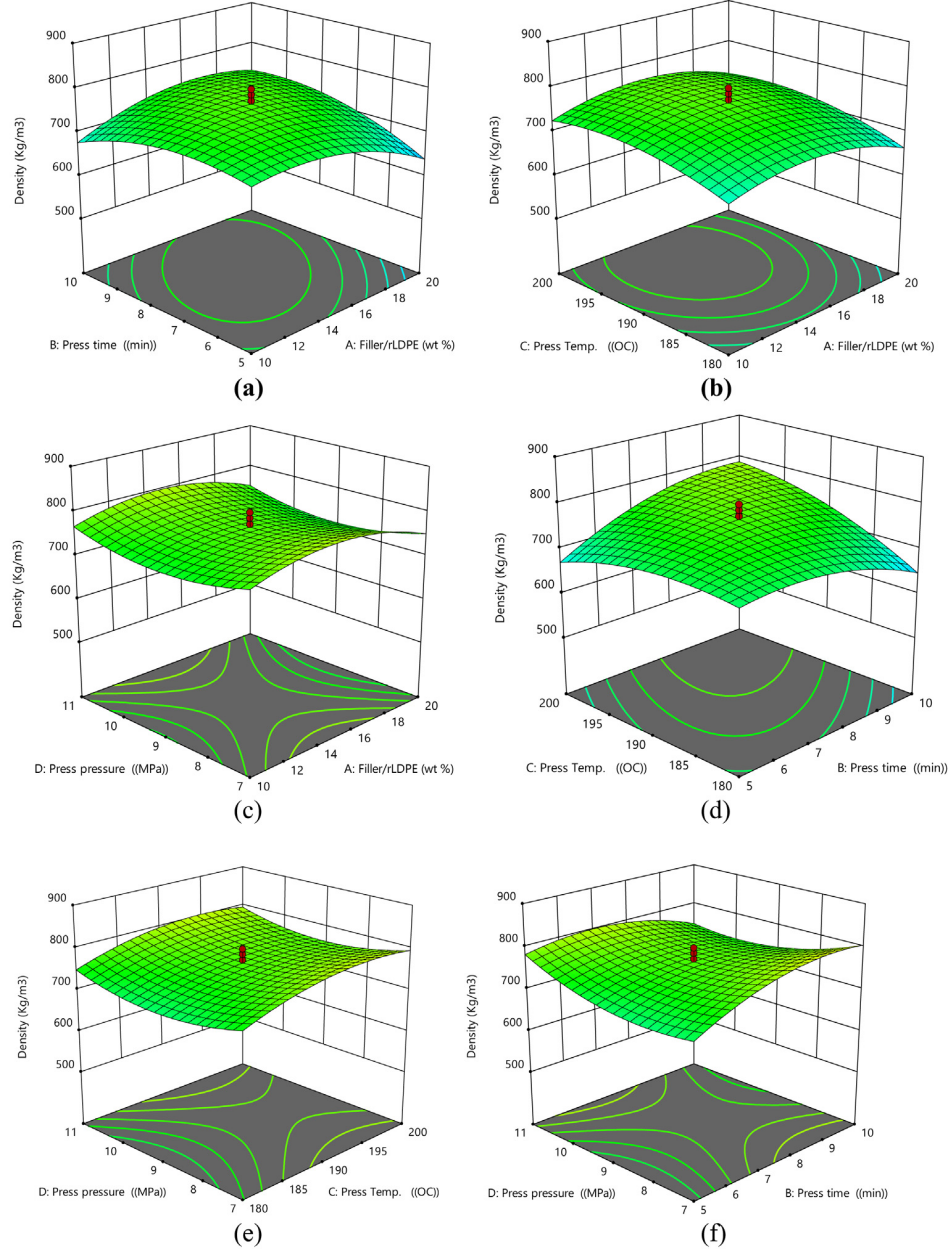
(a) 3D Plot of Press Time vs Filler/rLDPE on Density, (b) 3D Plot of Press Temperature vs Filler/rLDPE on Density, (c) 3D Plot of Press Temperature vs Press Time on Density, (d) 3D Plot of Press Pressure vs Filler/rLDPE on Density, (e) 3D Plot of Press Pressure vs Press Time on Density, (f) 3D Plot of Press Pressure vs Press Temperature on Density.

Fig 2a–f show the 3D surface plots for density. It indicates that density increases with increasing press time and filler/rLDPE ratio (Fig. 2a), density decreases with increasing filler/rLDPE and press temperature (Fig. 2b), density increases with increasing press pressure and filler/rLDPE (Fig. 2c), increasing press temperature and press time increases the density of the material (Fig. 2d), density decreases as the press pressure and press temperature decreases (Fig. 2e) and increasing press pressure and press time increases the density of the material (Fig. 2f).

### 3.2 Effect of the factors on water absorption

#### 3.2.1 ANOVA for water absorption

The **Model F-value** of 62.64 implies the model is significant. There is only a 0.01% chance that an F-value this large could occur due to noise. The **Lack of Fit F-value** of 3.01 implies the Lack of Fit is not significant relative to the pure error. There is a 11.79% chance that a Lack of Fit F-value this large could occur due to noise.

The final equation for water absorption gave;

(5) Water Absorption = 379.45831 + 10.52567X_1_ + 1.94909X_2_ − 4.74561X_3_ − 0.298673X_1_X_2_ − 0.055779X_1_X_3_ + 0.070181X_1_X_4_ − 0.036410X_3_X_4_ + 0.081762X_1_^2^ + 0.014346X_3_^2^

#### 3.2.2 3D surface plots for water absorption

**Fig. 3:**
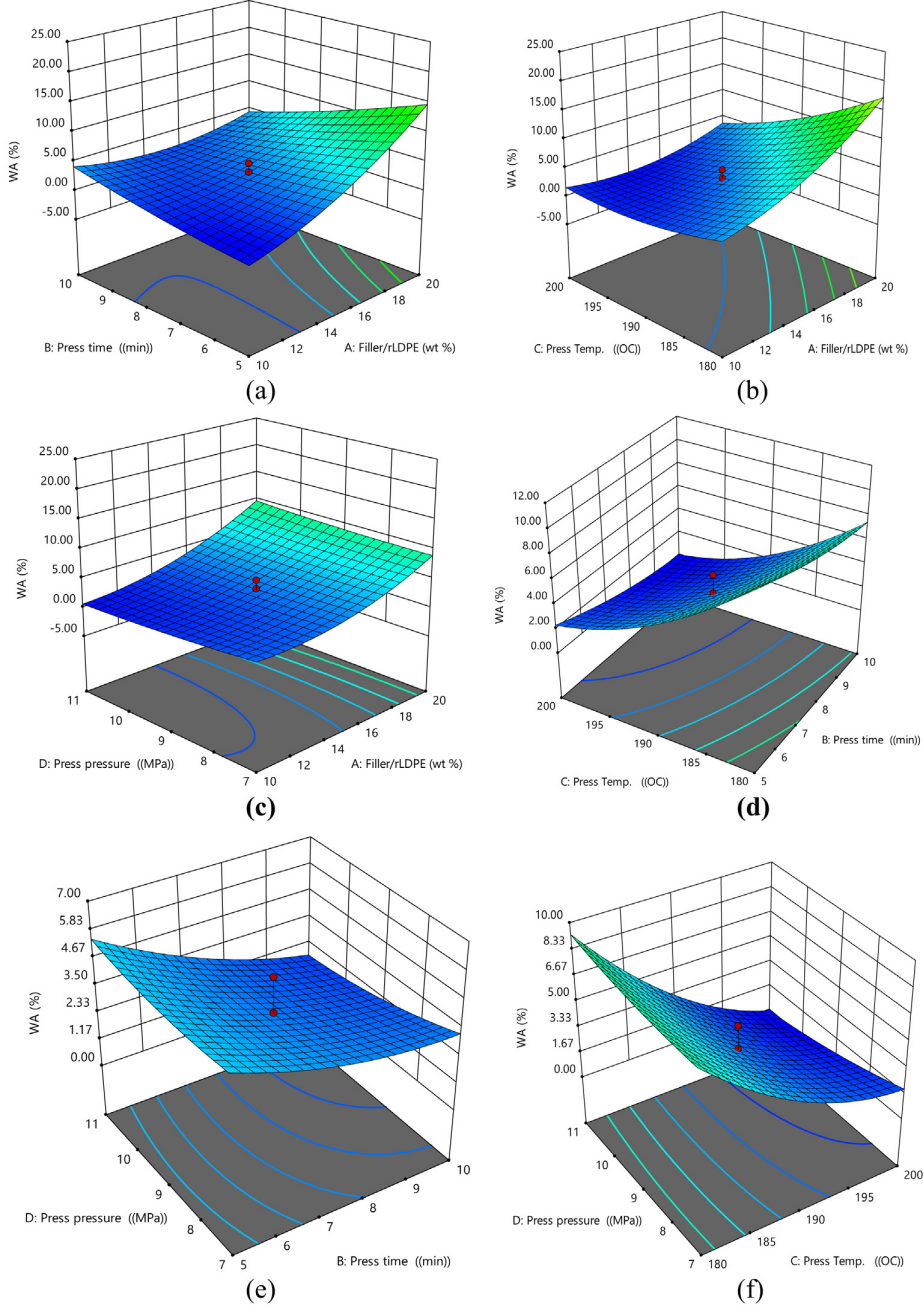
(a) 3D Plot of Press Time vs Filler/rLDPE on Water absorption, (b) 3D Plot of Press Temperature vs Filler/rLDPE on Water absorption, (c) 3D Plot of Press Temperature vs Press Time on Water absorption, (d) 3D Plot of Press Pressure vs Filler/rLDPE on Water absorption, (e) 3D Plot of Press Pressure vs Press Time on Water absorption, (f) 3D Plot of Press Pressure vs Press Temperature on Water absorption.

Fig 3a–f shows the surface plots for water absorption. It indicates that increasing press time and filler/rLDPE ratio will reduce the water absorption (Fig. 3a), increasing press temperature and filler/rLDPE ratio will cause a reduction in water absorption (Fig. 3b), increasing the press time and filler/rLDPE ratio will cause a corresponding decrease in the water absorption (Fig. 3c), increasing the press time and press temperature reduces water absorption (Fig. 3d), increasing press pressure and press time reduces the water absorption (Fig. 3e) and water Absorption decreases as the press pressure and press temperature decreases (Fig. 3f).

### 3.3 Effect of the factors on thickness swell

#### 3.3.1 ANOVA for quadratic model thickness swell

The **Model F-value** of 27.05 implies the model is significant. There is only a 0.01% chance that an F-value this large could occur due to noise. The **Lack of Fit F-value** of 1.39 implies the Lack of Fit is not significant relative to the pure error. There is a 37.70% chance that a Lack of Fit F-value this large could occur due to noise.

The final equation gave;

(6) Thickness Swell = −472.49411 + 3.62199X_3_ − 0.198972X_1_X_2_ − 0.046724X_2_X_3_ − 0.173914X_3_X_4_ − 0.044729X_1_^2^ − 0.181235X_2_^2^ + 1.37286X_4_

#### 3.3.2 3D surface plots for thickness swell

**Fig. 4:**
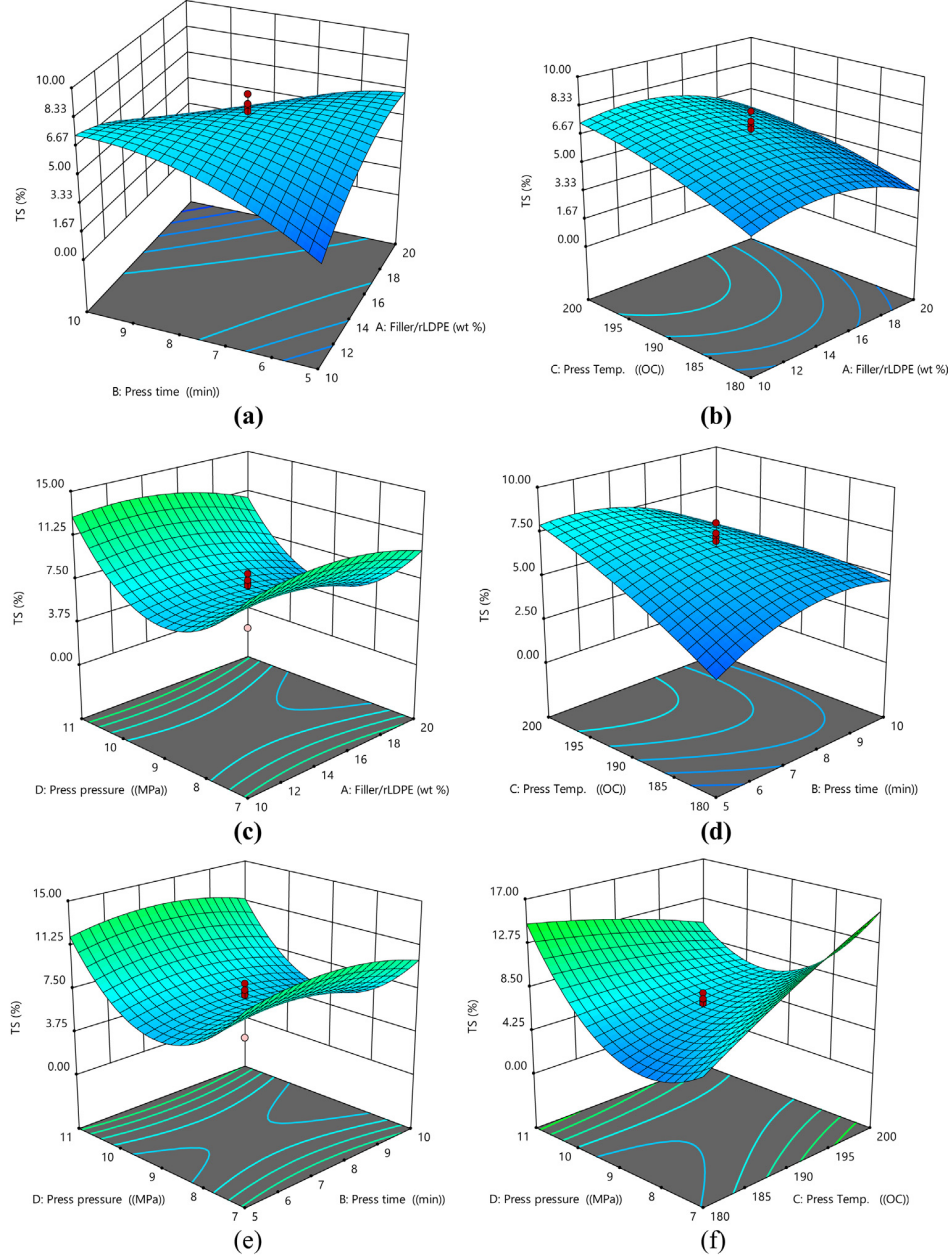
(a) 3D Plot of Press Time vs Filler/rLDPE on Thickness swell, (b) 3D Plot of Press Temperature vs Filler/rLDPE on Thickness swell, (c) 3D Plot of Press Temperature vs Press Time on Thickness swell, (d) 3D Plot of Press Pressure vs Filler/rLDPE on Thickness swell, (e) 3D Plot of Press Pressure vs Press Time on Thickness swell, (f) 3D Plot of Press Pressure vs Press Temperature on Thickness swell.

Fig. 4a–f shows the 3D surface plot for thickness swell. The plots indicate that increasing press time and filler/rLDPE ratio causes a reduction in thickness swell (Fig. 4a), thickness swell decreases as the press temperature decreases with a corresponding increase in filler/rLDPE ratio (Fig. 4b), as the filler/rLDPE and press pressure reduced the thickness reduces. At a pressure of 9MPa the thickness started increasing suggesting that a pressure of 9MPa should not be exceeded for press pressure and filler/rLDPE ratio combination (Fig. 4c), increasing the temperature and press time reduces the thickness swell of the material (Fig. 4d), increasing the press pressure and press time reduces the thickness (Fig. 4e), increasing pressure and press temperature causes a reduction in the thickness swell (Fig. 4f).

### 3.4 Effect of the factors on thermal conductivity

#### 3.4.1 ANOVA for quadratic model thermal conductivity

The **Model F-value** of 26.07 implies the model is significant. There is only a 0.01% chance that an F-value this large could occur due to noise. The **Lack of Fit F-value** of 0.37 implies the Lack of Fit is not significant relative to the pure error. There is a 91.59% chance that a Lack of Fit F-value this large could occur due to noise. Non-significant lack of fit is good -- we want the model to fit.

The final equation for thermal conductivity is;

(7) Thermal Conductivity = 45.62691 – 0.640298X_2_ − 0.368579X_3_ − 1.21297X_4_ + 0.003071X_1_X_4_ + 0.002312X_2_X_3_ + 0.004520X_3_X_4_ + 0.002366X_1_^2^ + 0.010906X_2_^2^ + 0.000775X_3_^2^ + 0.013111X_4_^2^

#### 3.4.2 3D surface plots for thermal conductivity

**Fig. 5:**
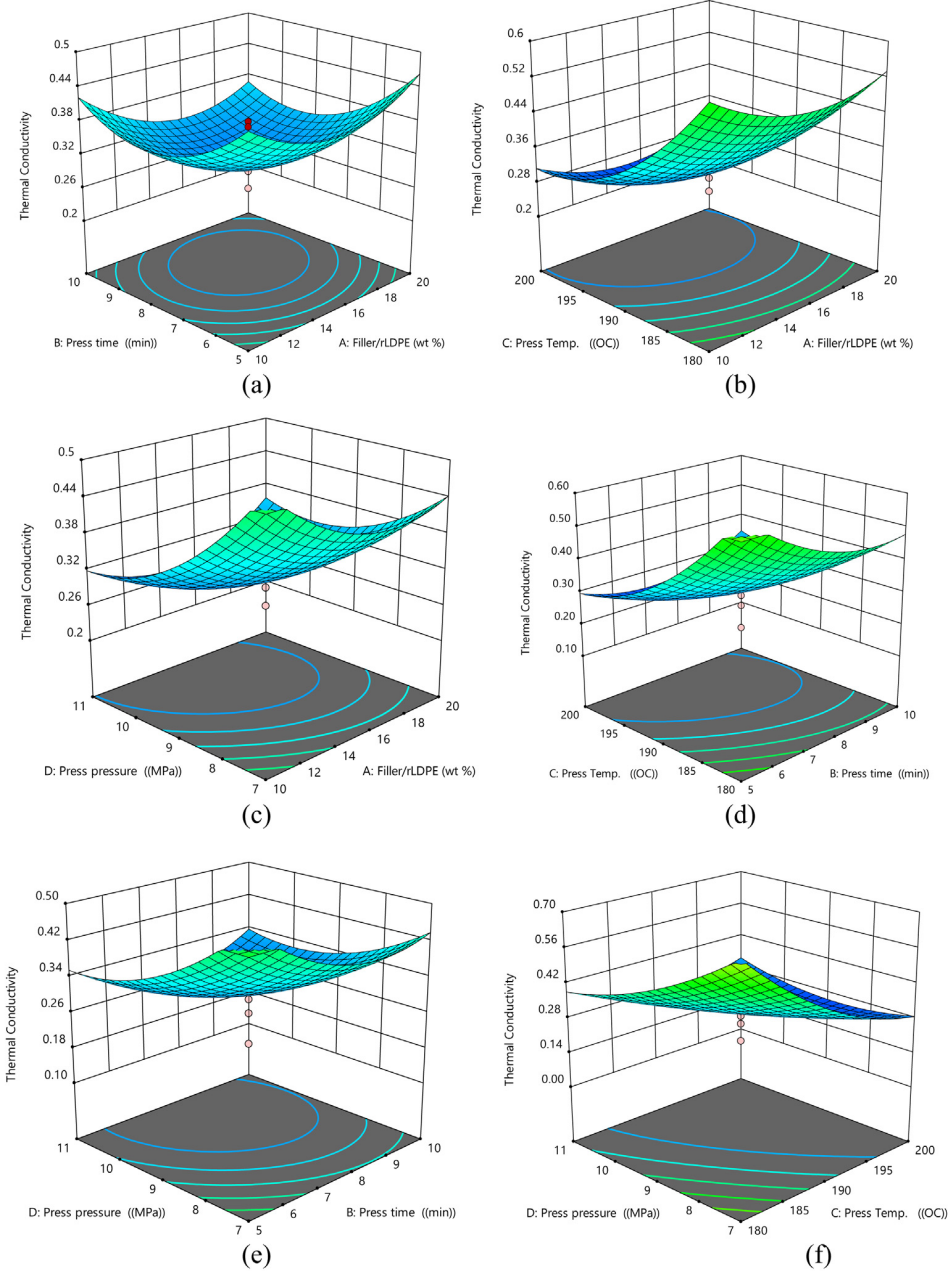
(a) 3D Plot of Press Time vs Filler/rLDPE on Thermal conductivity, (b) 3D Plot of Press Temperature vs Filler/rLDPE on Thermal Conductivity, (c) 3D Plot of Press Temperature vs Press Time on Thermal Conductivity, (d) 3D Plot of Press Pressure vs Filler/rLDPE on Thermal Conductivity, (e) 3D Plot of Press Pressure vs Press Time on Thermal Conductivity, (f) 3D Plot of Press Pressure vs Press Temperature on Thermal Conductivity.

Fig. 5a–f shows the 3D surface plot for thermal conductivity of the material. It indicates that increasing the press time and increase in filler/rLDPE ratio causes a reduction in the thermal conductivity of the material (Fig. 5a), thermal conductivity reduces as press time and filler/rLDPE ratio and press increases (Fig. 5b), thermal conductivity as press pressure and filler/rLDPE increases (Fig. 5c), increasing press time and press preasure causes a reduction in the thermal conductivity of the material (Fig. 5d), increasing press time and press pressure causes reduction in the thermal conductivity of the material (Fig. 5e) and increases press time and press pressure causes reduction in the thermal conductivity of the material (Fig. 5f).

### 3.5 Optimization solutions

The optimization tool gave ten solutions from which one was selected.

Putting the constraints, the software gave an optimal values of 19.7% filler/rLDPE, 10minutes press time, 197.31 °C and 9.042MPa press pressure.

### 3.6 Discussion of results

The optimisation tool of the design expert software gave the optimal composition of the constituents as well as the production parameters of the ceiling board. This implies that a mixture of 19.7% of bread fruit seed coat and 80.3% recycled low density polyethylene, compacted using a constant temperature hydraulic press at a temperature 197 °C and 9.04MPa for 10minutes will give a ceiling board of 775.661 g/cm^3^ density, 0.308% Water Absorption, 0.962% Thickness Swell and 0.367W/M.K Thermal Conductivity. The result gave a low thermal conductivity which makes the produced ceiling board a good thermal insulator. The low thermal conductivity is good property that will condition the temperature of the room.

A water absorption rate as low as 0.308% is good for a ceiling board in cases of leaking roofing sheet. Ceiling boards with high water absorption will definitely not last long in cases of leaking roofing sheets. The low thickness swell also means that the produced ceiling board will experience very little deformation when it comes in contact with moisture. When the obtained result is compared with those of existing ceiling, the new formulation is seen to compare favorably as shown in Table 10.

**Table 10:** Properties of New formulation and other existing ceiling boards.

Properties	Waste paper	Rice Husk Based	New formulation (Breadfruit seed coat based)
Thermal conductivity (W/M.K)	0.07-0.082 (Ataguba, 2016)	0.092 (Obam, 2012)	0.367
Water absorption rate (%)	-	14.5 (Ataguba, 2016)	0.308
Density (g/cm^3^)	415 (Ekpunobi et al., 2015)	745-1022 (Idehai, 2012)	775.661
Thickness swell (%)	**-**	**-**	0.962

## 4 Conclusion

From this research it can be concluded that breadfruit seed coat is a good filler material when combined with recycled low density polyethylene. For desired physical and mechanical properties like thermal conductivity, water absorption, thickness swell and density, production parameters which include filler/rLDPE ratio, press time, press temperature and press pressure must be put to check. These production parameters were optimized in this research using Response Surface methodology. The optimized values obtained are; 19.722% filler/rLDPE, 10minuttes press time, 197.31 °C and 9.042MPa press pressure. These values gave the produced ceiling physical and mechanical characteristics of 775.661 g/cm^3^ density which is close to that of jatropha curcas seedcake based with a density of 897.5 g/cm^3^ developed by Olorunmaiye and Ohijeagbon (2015). The obtained density falls within the range of 745–1022 g/cm^3^, for rice husk based ceiling board reported by Idehai (2012).

The obtained Water Absorption rate 0.308% is far better than that of 7.5% and 14.5%, for waste paper and rice husk based ceiling board, reported by Ataguba (2016). This gave a density of 775.661 g/cm^3^, water absorption of 0.308%, thickness swell of 0.962%, and a thermal conductivity of 0.367W/m.K. With this, the developed material can compete favorably withe the existing ceiling boards. Obam (2012) also reported a thermal conductivity of 9.2 × 10^−2^W/mK, for sawdust, paper and starch based ceiling board, this however appears to have a better thermal insulating property than the developed breadfruit seed coat based ceiling board.

Hence the results obtained in comparison to other agro and synthetic waste based ceiling board, makes the developed breadfruit seed coat and recycled low density polyethylene based ceiling a good option for buildings.

## Declarations

### Author contribution statement

Ezenwa O.: Conceived and designed the experiments; Wrote the paper.

Obika E. N.: Performed the experiments; Wrote the paper.

Umembamalu C.: Analyzed and interpreted the data; Wrote the paper.

Nwoye F. C.: Contributed reagents, materials, analysis tools or data; Wrote the paper.

### Funding statement

This research did not receive any specific grant from funding agencies in the public, commercial, or not-for-profit sectors.

### Competing interest statement

The authors declare no conflict of interest.

### Additional information

No additional information is available for this paper.
